# Supplementary material for: Conditional chemoconnectomics (cCCTomics) as a strategy for efficient and conditional targeting of chemical transmission
Source: eLife. 2024 Apr 30;12:RP91927. doi: 10.7554/eLife.91927 (PMC11060718; doi:10.7554/eLife.91927)
Supplement: Figure 3—source data 3. [file elife-91927-fig3-data3.zip › Figure 3 source data3 part 2.zip!/Figure 3-Source data 3-part2/1052-13-CRISPResso_Report_ejedac2.zip!/CRISPResso_on_1052_16/9.Alleles_frequency_table_around_sgRNA_agtggacaaactggtacatg.pdf]

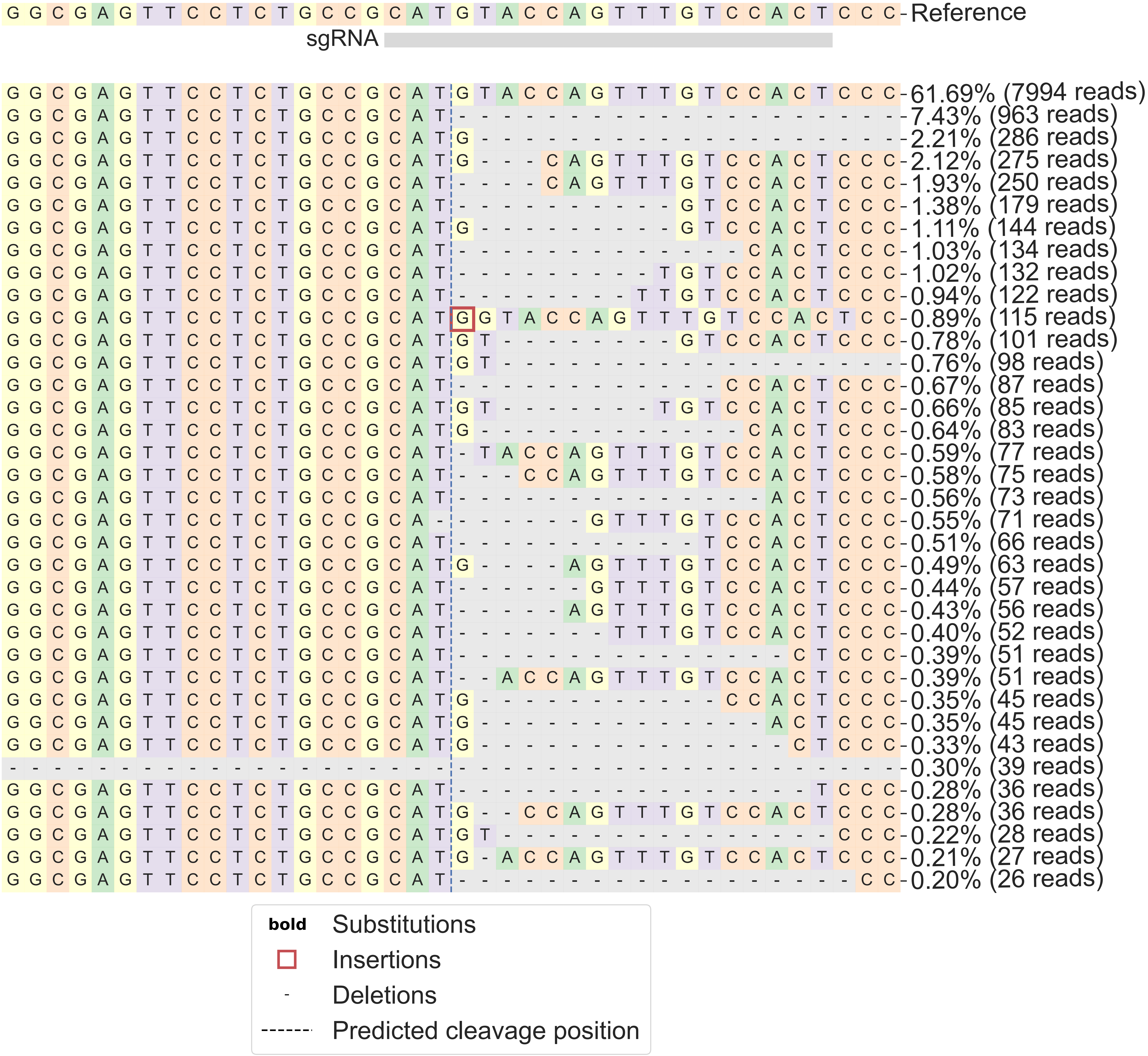
GGCGAGTTCCTCTGCCGCATGTACCAGTTTGTCCACTCCC Reference
sgRNA
GGCGAGTTCCTCTGCCGCATGTACCAGTTTGTCCACTCCC 61.69% (7994 reads)
GGCGAGTTCCTCTGCCGCAT-------------------- 7.43% (963 reads)
GGCGAGTTCCTCTGCCGCATG------------------- 2.21% (286 reads)
GGCGAGTTCCTCTGCCGCATG---CAGTTTGTCCACTCCC 2.12% (275 reads)
GGCGAGTTCCTCTGCCGCAT----CAGTTTGTCCACTCCC 1.93% (250 reads)
GGCGAGTTCCTCTGCCGCAT----------GTCCACTCCC 1.38% (179 reads)
GGCGAGTTCCTCTGCCGCATG---------GTCCACTCCC 1.11% (144 reads)
GGCGAGTTCCTCTGCCGCAT-------------CACTCCC 1.03% (134 reads)
GGCGAGTTCCTCTGCCGCAT---------TGTCCACTCCC 1.02% (132 reads)
GGCGAGTTCCTCTGCCGCAT--------TTGTCCACTCCC 0.94% (122 reads)
GGCGAGTTCCTCTGCCGCAT G GTACCAGTTTGTCCACTCC 0.89% (115 reads)
GGCGAGTTCCTCTGCCGCATGT--------GTCCACTCCC 0.78% (101 reads)
GGCGAGTTCCTCTGCCGCATGT------------------ 0.76% (98 reads)
GGCGAGTTCCTCTGCCGCAT------------CCACTCCC 0.67% (87 reads)
GGCGAGTTCCTCTGCCGCATGT-------TGTCCACTCCC 0.66% (85 reads)
GGCGAGTTCCTCTGCCGCATG------------CACTCCC 0.64% (83 reads)
GGCGAGTTCCTCTGCCGCAT-TACCAGTTTGTCCACTCCC 0.59% (77 reads)
GGCGAGTTCCTCTGCCGCAT---CCAGTTTGTCCACTCCC 0.58% (75 reads)
GGCGAGTTCCTCTGCCGCAT--------------ACTCCC 0.56% (73 reads)
GGCGAGTTCCTCTGCCGCA-------GTTTGTCCACTCCC 0.55% (71 reads)
GGCGAGTTCCTCTGCCGCAT-----------TCCACTCCC 0.51% (66 reads)
GGCGAGTTCCTCTGCCGCATG----AGTTTGTCCACTCCC 0.49% (63 reads)
GGCGAGTTCCTCTGCCGCAT------GTTTGTCCACTCCC 0.44% (57 reads)
GGCGAGTTCCTCTGCCGCAT-----AGTTTGTCCACTCCC 0.43% (56 reads)
GGCGAGTTCCTCTGCCGCAT-------TTTGTCCACTCCC 0.40% (52 reads)
GGCGAGTTCCTCTGCCGCAT---------------CTCCC 0.39% (51 reads)
GGCGAGTTCCTCTGCCGCAT--ACCAGTTTGTCCACTCCC 0.39% (51 reads)
GGCGAGTTCCTCTGCCGCATG-----------CCACTCCC 0.35% (45 reads)
GGCGAGTTCCTCTGCCGCATG-------------ACTCCC 0.35% (45 reads)
GGCGAGTTCCTCTGCCGCATG--------------CTCCC 0.33% (43 reads)
-- 0.30% (39 reads)
GGCGAGTTCCTCTGCCGCAT----------------TCCC 0.28% (36 reads)
GGCGAGTTCCTCTGCCGCATG--CCAGTTTGTCCACTCCC 0.28% (36 reads)
GGCGAGTTCCTCTGCCGCATGT---------------CCC 0.22% (28 reads)
GGCGAGTTCCTCTGCCGCATG-ACCAGTTTGTCCACTCCC 0.21% (27 reads)
GGCGAGTTCCTCTGCCGCAT------------------CC 0.20% (26 reads)
bold Substitutions
Insertions
Deletions
Predicted cleavage position
